# Facilitators of HCV treatment adherence among people who inject drugs: a systematic qualitative review and implications for scale up of direct acting antivirals

**DOI:** 10.1186/s12889-016-3671-z

**Published:** 2016-09-20

**Authors:** Zachary C. Rich, Carissa Chu, Jessica Mao, Kali Zhou, Weiping Cai, Qingyan Ma, Paul Volberding, Joseph D. Tucker

**Affiliations:** 1 UNC Project China, Guangdong Provincial Dermatovenerology Hospital, Guangdong Province, 2 Lujing Road, Floor 11, Guangzhou, 510095 Guangdong China; 2 Department of Medicine, School of Medicine, University of California San Francisco, San Francisco, 94143 California USA; 3 Guangzhou Number Eight People’s Hospital, Guangzhou, 510000 China; 4 Institute of Global Health and Infectious Diseases, University of North Carolina at Chapel Hill, Chapel Hill, 27517 North Carolina USA

**Keywords:** Hepatitis C virus, Injection drug use, Treatment adherence

## Abstract

**Background:**

While the public health benefits of new HCV treatments depend on treatment adherence, particularly among people who inject drugs (PWID), several social and medical factors can jeopardize treatment adherence. The aim of this study is to examine the qualitative literature on facilitators to HCV treatment adherence among PWID.

**Methods:**

We searched six databases to identify qualitative research studies on HCV treatment adherence facilitators among PWID. Two reviewers independently extracted and analyzed data using PRISMA guidelines and the CASP tool to evaluate study quality.

**Results:**

From ten studies representing data from 525 participants, three major themes emerged across studies: logistical facilitators within health systems enhanced HCV treatment adherence, positive social interactions between PWID and staff provided positive feedback during treatment, and HCV treatment may complicate the addiction recovery process.

**Conclusions:**

Although PWID face several barriers to adherence, we identified treatment adherence facilitators that could be incorporated into clinical practice.

**Electronic supplementary material:**

The online version of this article (doi:10.1186/s12889-016-3671-z) contains supplementary material, which is available to authorized users.

## Background

Hepatitis C virus (HCV) remains a significant cause of morbidity and mortality, affecting over 130 million people worldwide [1]. HCV is estimated to be responsible for roughly one quarter of all cases of hepatocellular carcinoma and cirrhosis, [2] and mortality from HCV-related liver disease has surpassed mortality from HIV in the United States [3]. HCV is more commonly transmitted through blood transfusions and iatrogenically in developing countries, and through intravenous drug use (IVDU) in developed countries, though this remains an issue in developing countries as well [4]. In general, the burden of HCV infection falls disproportionately on people who currently inject or have injected drugs in the past (PWID), with HCV antibodies reported in greater than 60 % of PWID in 37 of 77 geographically and economically dispersed countries [5]. Unfortunately, PWID are frequently excluded from treatment due to concerns about adherence, concurrent drug use, psychosocial comorbidities and the higher risk of reinfection secondary to active drug use [6, 7]. Studies have shown that the likelihood of achieving sustained virological response (SVR) is much higher when patients are adherent to treatment, [8] and that there is little difference in SVR rates between PWID and others [9]. While prior research has focused on facilitators to testing and initiating treatment among PWID, [10] little is known about facilitators that specifically help this vulnerable population achieve high levels of adherence.

For decades, interferon-based therapy has been the standard of care for HCV, most popularly a regimen of pegylated interferon and ribavirin (pegIFN + RBV). However, long treatment courses, hematologic and psychiatric side effects, and the uncertainty of achieving SVR complicate interferon-based treatment for many patients and providers [11, 12]. Even for patients who pass screening for treatment, implementation can be challenging. The HCV care continuum involves multiple steps—serologic testing, radiographic imaging, possible biopsy, attendance at multiple follow-up visits, and adherence to a demanding treatment regimen [13]. Minor setbacks, both physiological, such as difficult to handle side effects, and non-physiological such as logistical difficulties, at any point may jeopardize the success of treatment.

Recent advances in the treatment of HCV in the form of direct acting antivirals (DAAs) will broaden treatment eligibility and options. DAAs may be used in conjunction with pegIFN + RBV, but interferon-free DAA regimens have reduced side effect profiles while still achieving SVR rates as high as 90 % with a shorter treatment duration: 8–12 weeks compared to several months with interferon therapy [13–15]. All of these factors help make treatment more tolerable, especially among patients with comorbid mental health disease or substance abuse issues [16]. In the United States and other high-income countries, treatment with DAAs is now the standard of care, though the high cost (over $100,000 USD per treatment course in the United States) presents a significant barrier in low- and middle-income countries (LMIC) [4]. Likely reflecting the difficulties in global access to DAA, the WHO’s guidelines for management of hepatitis C released in 2014 continued to prioritize pegIFN + RBV treatments worldwide [13]. However, as DAAs become the standard of treatment, both their long-term success and avoidance of drug resistance will hinge on achieving high levels of adherence [17]. Given the disproportionately high burden of disease among PWIDs, it is imperative that they are included in DAA scale-up programs and achieve high levels of adherence [18].

Qualitative data provide an important perspective on adherence for three reasons. First, adherence may directly depend on social factors that are not captured in quantitative studies. Second, qualitative data can provide rich information about values, preferences, and implications for human rights that are not included within randomized controlled trials. Finally, qualitative reviews of adherence in tuberculosis [19] HIV [20] and other diseases [21, 22] have provided useful information. The purpose of this systematic review is to evaluate qualitative studies investigating facilitators to HCV treatment adherence among PWID.

## Methods

The protocol for this study was registered on PROSPERO, the International Prospective Register of Systematic Reviews (CRD42013006057).

### Search strategy and selection criteria

Six electronic databases (CINAHL, ACS, MEDLINE, EMBASE, PSYCInfo, PubMed) were searched for citations related to treatment adherence in HCV-positive PWID. More detail on the search algorithm is included in Additional file 1. Studies were included if they were qualitative, written in the English language, and published in a peer-reviewed journal. We followed guidance from PRISMA [23].

**Fig. 1:**
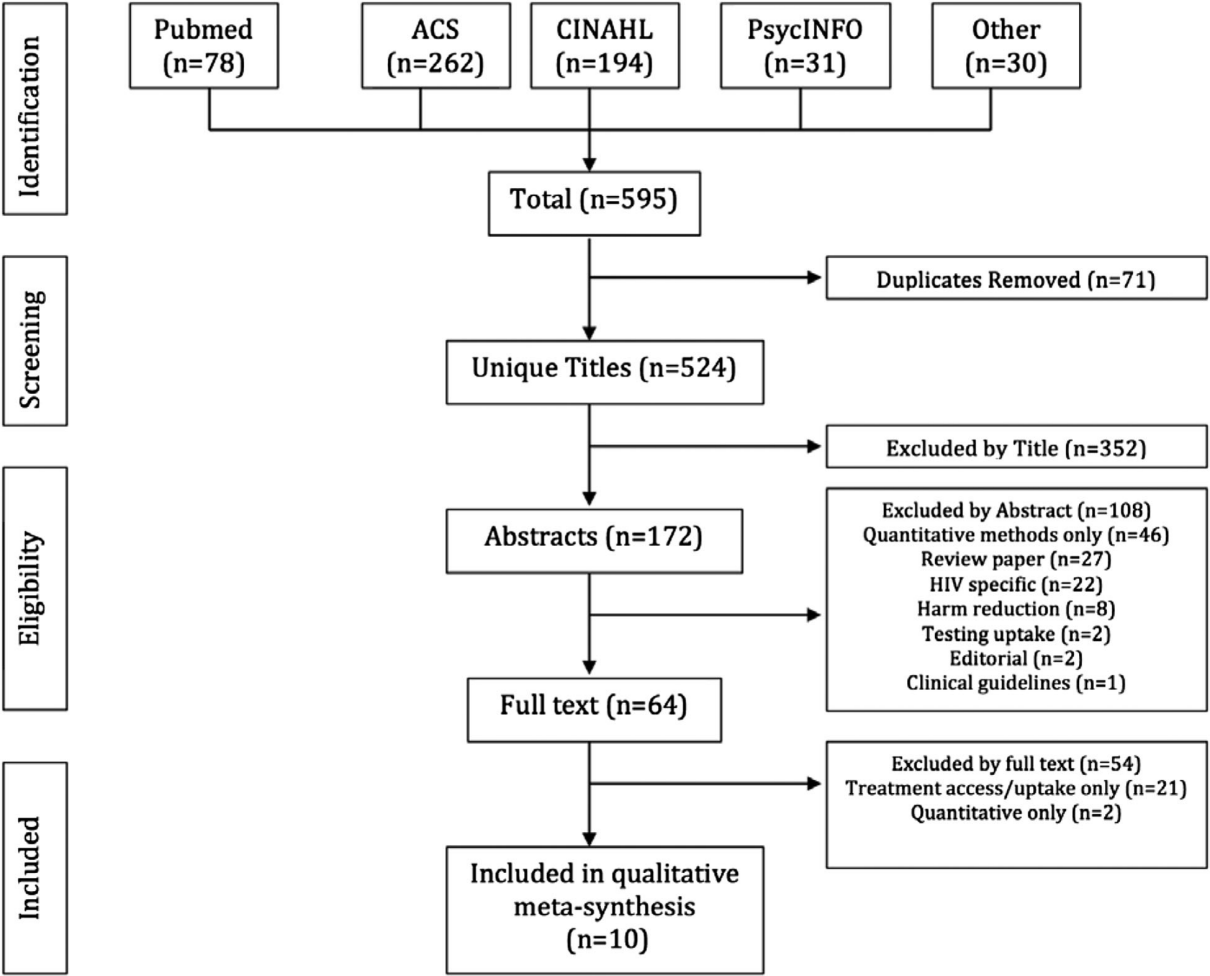
Flowchart demonstrating search strategy

Qualitative methods accepted included in-depth interviews, focus groups, personal narratives, and mixed method studies. The search was initially completed February 20, 2014 and updated last on October 27, 2015. Studies on current and former injection drug users with HCV, including those in methadone treatment, were included. While our study focused on HCV medication adherence, qualitative reviews also covering HCV linkage and retention in care were included if relevant. Studies dealing with HIV/HCV co-infected patients were included, if applicable. Titles and abstracts were evaluated for relevance by a single independent reviewer. Full texts were evaluated by two independent reviewers. Discrepancies were brought to a third independent reviewer for discussion and resolution. Detailed study screening methods and a search flow diagram according to PRISMA guidelines are included (Fig. 1).

### Analysis and synthesis

The objective of this meta-synthesis was to identify facilitators to HCV treatment adherence among HCV-infected PWID. We utilized the Noblit and Hare [24] meta-ethnographic approach that has been widely used for other medical qualitative systematic reviews [25]. Findings from one citation were compared with findings from another paper through reciprocal translation then condensed to create thematic structures. Each paper was systematically reviewed by two authors for missing data and data not encompassed in the thematic structures. Themes were then used to summarize facilitators of HCV adherence among injection drug users. Participant identifiers were included in the results when they were present in the primary data.

### Study appraisal

After a final list of full text articles was obtained, three authors (ZCR, CC and JM) independently reviewed all included studies and assessed them for completeness using the Consolidated Criteria for Reporting Qualitative Research (COREQ) checklist [26], a standardized and way to evaluate qualitative research studies. Included studies were then evaluated using the Critical Appraisal Skills Programme (CASP) tool (Table 1).

**Table 1:** Quality assessment of included research studies

First Author (Year)	Is the study qualitative research?	Is the study context clearly described?	Is there evidence of researcher reflexivity?	Is the sampling method clearly described and appropriate for the research question?	Is the method of data collection clearly described and appropriate to the research question?	Is the method of analysis clearly described and appropriate to the research question?	Are the claims made supported by sufficient evidence? i.e., did the data provide sufficient depth, detail, and richness?	Total
Harris (2009) [47]	1	1	1	1	1	0	1	6
Harris (2013) [35]	1	1	1	1	1	1	1	7
Hopwood (2007) [29]	1	1	1	1	1	1	1	7
Munoz Plaza (2008) [30]	1	1	1	1	1	1	1	7
Nguyen (2007) [31]	1	1	0	1	1	0	1	5
Norman (2008) [32]	1	1	1	1	1	1	1	7
Strauss (2005) [12]	1	1	1	1	1	1	1	7
Treloar (2008) [27]	1	1	1	1	1	1	1	7
Treloar (2013) [33]	1	1	1	1	1	1	1	7
Rasi (2014) [34]	1	1	1	1	1	1	1	7

## Results

Our search yielded a total of 595 citations of which ten met inclusion criteria. Figure 1 details the exclusion of papers at each stage of the screening process.

### Description of studies

All ten studies were conducted in high-income, Western countries. One study examined adherence to interferon only, [27] eight studies examined interferon and ribavirin regimens [10, 12, 28–33] and one study examined DAA triple therapy adherence [34]. A description of these studies can be found in Table 2. These studies represent data from 525 participants and were conducted through in-depth interviews, [10, 12, 27–30, 32–34] focus groups, [12, 30, 32] and personal narratives [31]. The number of participants in each study ranged from 2 to 164.

**Table 2:** Manuscripts included in qualitative evidence review (*n* = 814 individuals in 18 studies)

First author	Year	Location	Study design	Sample size (*n*)	Sample population	Treatment methods
Harris	2009	Australia	Interviews^a^	40	Former PWID seeking care	Interferon + ribavirin
Harris	2013	United Kingdom	Interviews	49	Current and former PWID and providers	Interferon + ribavirin
Hopwood	2007	Australia	Interviews	20	Patients and providers from a HCV treatment center	Interferon-based, unspecific
Munoz Plaza	2008	USA	Interview, Focus groups	164	Patients enrolled in drug treatment programs	Interferon + ribavirin (not all patients on treatment)
Nguyen	2007	Australia	Personal narratives	3	Patients enrolled in PEG-IFN treatment study	Interferon OR interferon + ribavirin if HIV/HCV co-inf
Norman	2008	Australia	Interviews, Focus groups	10	Current or former PWID seeking care or working at HCV treatment center	Interferon + ribavirin
Strauss	2005	USA	Interviews, Focus groups	72	Patients and staff in a drug treatment programs	Interferon + ribavirin
Treloar	2008	Australia	Interviews	77	Current and former drug treatment patients	Interferon only (*n* = 2)
Treloar	2013	Australia	Interviews	76	HCV patients receiving opiate-substitution treatment	Interferon + ribavirin
Rasi	2014	Switzerland	Interviews	14	HCV patients undergoing protease-inhibitor therapy	DAA triple therapy (Protease inhibitor, interferon, ribavirin)

Abbreviations: *PWID* people who inject drugs, *HCV* hepatitis C virus, *PEG-IFN* pegylated interferon

^a^Interviews refer to one-on-one, in-depth interviews

### Description of the themes

The emergent themes are shown in Table 3. We identified three overarching final themes: logistical support for patients; positive interactions with support staff; and understanding the drug user identity.

**Table 3:** Initial concepts, emerging themes, and final themes related to HCV treatment adherence facilitators

Initial concept	Relevant papers	Emerging themes	Final themes
Shelter assistance	Strauss 2005	Support outside the clinic	Logistical support for patients
Transportation assistance	Harris 2013, Norman 2008, Rasi 2014		
Integration of services	Norman 2008, Strauss 2005, Harris 2013, Treloar 2013	Accommodating clinic	
Flexible appointment times	Rasi 2014		
Accommodating phlebotomy services	Harris 2013		
Kind and attentive care	Harris 2013, Norman 2008, Strauss 2005, Treloar 2013, Rasi 2014	Compassionate clinical staff	Positive interactions with support system
Peers who understand addiction	Norman 2008, Strauss 2005	Support of current and former injection drug users	
Examples of treatment success	Munoz-Plaza 2008, Treloar 2013		
Avoid needles that trigger relapse	Harris 2009, Nguyen 2007	Help avoiding substance abuse relapse	Understanding drug user identity
Managing side effects that mimic withdrawal	Harris 2009, Strauss 2005		
Improving health to care for others	Treloar 2013		

### Theme one: logistical support for patients

Many patients expressed that their complex financial and medical needs must concurrently be accounted for in order to maintain high levels of adherence. This theme included facilitators both outside of the health services (such as transportation and job assistance) and within the healthcare system (flexible clinic hours and integration of services).

#### Support outside the healthcare system: “I wouldn’t think of doing this if I was out on the street”

Transportation was mentioned frequently among patients as a critical mediator of adherence [32, 35–37]. Two studies provided transportation assistance for HCV-infected PWID to HCV clinical services (Additional file 2, Row1) [31, 32].

*“Well partly the transport [is a barrier], but I think it’s a psychological thing for a lot of our patients, they’re very entrenched in their own environment…. You tell them that you’ve got an appointment at [hospital name], well you might as well say it’s in Timbuktu because they have no idea where that is*” [35].

In addition to the physical act of getting a patient to their appointment, transportation services may be helpful for guiding patients to an unfamiliar treatment center (Additional file 2, R1) [35].

Additionally, services that provided long-term housing for patients undergoing treatment helped patients tolerate the side effects of interferon therapy, as lack of stable environment was cited as a barrier to remaining adherent to treatment (Additional file 2, Row2) [38].

For homeless patients, having access to shelter services following the weekly interferon injections was seen as necessary for treatment completion (Additional file 2, Row3) [12].

*“I wouldn’t think of doing this if I was out on the street… I mean, I used to be in the bed after taking that stuff, just shaking… I would never been able to go through that out on the street”* [12].

#### Accommodating clinic: “My needs are met in a whole lot of different ways, from personal to support, to my addiction to ramifications from the addiction”

Many participants highlighted logistical factors within the clinic that facilitated their ongoing treatment. Paramount among these was the integration of HCV services with other services that are commonly sought by PWID such as addiction specialists, mental health services, and other general medical services (Additional file 2, Row4) [32].

*“My needs are met in a whole lot of different ways, from personal to support, to my addiction to ramifications from the addiction”* [32].

This integration of services was seen as “making it easier” not having to “run around” [32]. Several studies commented on improving adherence through HCV treatment integration with inpatient [12, 37] or outpatient drug rehabilitation programs [32, 35, 38]. Patients often felt more comfortable receiving HCV treatment at drug treatment centers where they were less worried about treatment-associated stigma [33]. However, others were worried that receiving integrated services may threaten confidentiality, [33] though accounts of this happening were not reported in any study.

Participants also identified flexible clinic hours for receiving treatment, testing, or other services as another mediator of adherence [34]. This was seen as particularly true for patients with ongoing substance use.

Lastly, patients identified the importance of more accessible phlebotomy services. Patients felt that more convenient hours and well-trained, non-judgmental phlebotomists would improve their outlook on continuing with their treatment regimens [10].

*“I kept on saying to [hospital phlebotomist], ‘Look, you know, my veins are a nightmare, you know, let me do it’. [She said] ‘Oh you people, you think you know about your veins and all that, when you know nothing”* [35].

### Theme two: positive interactions with support system

Many studies referenced patients’ perceptions of their support systems, including attitudes from clinical staff as well as formal or informal support groups, such as other PWID.

#### Compassionate providers: “with this type of support I could continue taking this drug”

Both patients and providers noted that positive patient-provider relationships promoted treatment adherence [12, 29, 32, 37, 39] (Additional file 2, Row7) Providers who expressed concern and understanding gained the trust of patients to withstand treatment and made the side effects of the interferon more manageable [12, 35].

*“About 4 or 5 months in the program, I started taking the interferon. The people were very attentive to me; the nurses were great—with this type of support I could continue taking this drug”* [12].

Clinical staff familiar with PWID were preferable to general staff, particularly because of perceptions of respect between the patient and the staff [29, 37].

*“Just the people here. You can talk to ‘em a lot better. They don’t look down on you. They actually talk to you. They explain every- thing. You go to places, other places, you know, yeah, they don’t make you feel very welcome”* [33].

#### Support of current and former injection drug users: “if we didn’t have the peer support worker this program wouldn't be running”

In one study, a peer counselor model was critical in providing support throughout the treatment process [32]. Peer counselors were seen as understanding and approachable, and they served as cultural ambassadors between doctors and patients (Additional file 2, Row8) [32].

*“I think she has made the difference between sticking to this or not…I have had more times where I have thought ‘[expletive] this, I’m not doing this anymore, go shove it up your [expletive]’ but if she wasn’t here there would have been many more times. [I]f we didn’t have the peer support worker this program wouldn’t be running”* [32].

Peers served as examples of patients who had successfully undergone treatment and cleared HCV [30, 39]. Having a shared experience with other PWID supported patients through treatment. This was true for both organized support groups [32] and informal relationships [12]. Support groups, counseling sessions, and family support gave patients the opportunity to disclose drug use and treatment experience openly. In one study, a methadone client on HCV treatment described how important it was for her to know that “you’re not alone in this. Love and concern is here, and that plays a major part” [12].

Patients expressed desire to talk specifically with peers with first-hand experience with treatment, [30] finding a positive source of support in sharing challenges and successes. They felt that the more they learned about their disease from peers or providers, the more likely they were to continue with their HCV treatment [32].

### Theme three: understanding drug user identity

#### Avoiding relapse and managing ongoing drug use

Patients often stated that their first priority was to remain sober and that any treatment for HCV would have to be secondary to this goal. HCV treatment may complicate the recovery process for PWID concurrently engaged in addiction recovery [12, 40]. Patients may need to interrupt their attendance at addiction services and counseling, or physically leave a detoxification or rehabilitation setting to attend clinic appointments [12]. HCV treatment itself has been attributed to drug relapse due to the complex constellation of side effects of interferon treatment on mental health, further compounded by alcohol, drug use, and substitution pharmacotherapies [35, 41, 42].

*“The depression kicks in, and you are bed-ridden. It is hard enough just staying clean without it [HCV treatment]. That is the scary part”* [28].

Others noted that the side effects resembled opioid withdrawal [28, 41].

*“All my joints ached. I was in a sweat. I felt like I was hanging out [withdrawing from heroin]. I felt like I’d had a dirty hit…. I did think ‘a shot [of heroin] would make me feel better’”* [28].

Patients also stated that the act of injecting interferon was itself addicting [31] and acted as a “trigger” for wanting to use again (Additional file 2, Row9) [28].

Some facilitators mentioned were use of alternative interferon delivery devices that minimized resemblance to injecting heroin [28] and medications such as cannabis to minimize withdrawal like side effects of interferon (Additional file 2, Row11) [29].

*“Quite a few patients smoke cannabis … for relief of nausea, to help them sleep, to help with aches and pains, to help them relax. So, if they continue to do that, that’s fine. I just ask them to tell me how much they’re doing… because it can impact on mood”* [29].

For current PWID, clinic policies that did not insist on complete sobriety were a facilitator to treatment.

“*We’ve got one guy, he injects probably once a week and he comes for his appointments… he’s not trying to work towards not using, that’s just part of his life. I think it is important that people like him manage to access treatment, that it’s not an exclusion criteria*” [29].

#### Aspiring for new healthier identity: “on a good day I’m gonna feel like really brilliant”

Some studies identified that although some patients did not care for their own health, they would complete treatment for the sake of others [33].

HCV treatment was also seen by PWID as an opportunity to create a new image for themselves. Many patients saw HCV infection as a drug-related problem, and likened completion of HCV treatment to a last step to recovery.

*“You come in here to stop drinking and using drugs; I don’t want to die no more. I don’t want to go, to live in a box. I don’t want to eat out of the garbage. I don’t want to go to jail. I want to change”*[12].

## Discussion

This review explores ways to improve HCV treatment adherence among PWID and has implications for scaling up DAA treatment regimens. Better understanding PWID in the context of HCV treatment adherence will be critical for expanding DAA regimens. Several review papers have examined the social context of HCV treatment among PWID, [10, 43, 44] with a focus on access to treatment [10]. This review expands the literature by using a systematic approach (including formal assessment of study quality), focusing on facilitators of HCV adherence, and considering implications for DAA scale up.

Our findings suggest that integrating HCV treatment and addiction services will enhance HCV treatment adherence among PWID. Few quantitative studies have evaluated integration of these services as an intervention [45, 46]. These studies, though small in sample size, have shown promising results for integrated clinics. For example, one study showed treatment adherence among 86 % of patients in an integrated clinic, with adherence defined as completing ≥80 % of treatment regimen [47]. The process of integration ranges from services co-located in a single clinic [27, 38] to facilitated addiction service referrals at HCV treatment clinics and may even include a mix of these [41]. Integration would mitigate several logistical barriers that PWID commonly encounter [32, 35]. Addiction treatment and HCV treatment regimens are both physically [12] and emotionally demanding, [34] and patients report missing one at the expense of the other, [12] This problem is minimized through offering care for both at the same visit. Furthermore, PWID also report feeling more comfortable receiving care from doctors and nurses experienced in treating injection drug users, such as in drug treatment centers [33]. Concerns with integrated clinics include further linking HCV to intravenous drug use and increasing the stigma of the disease, [37] lack of preparation by addiction care providers to provide HCV care, [38] and patient reluctance to fully disclose drug-related information at the risk of disqualification from addiction care [48]. However, these concerns are theoretical, and have not been documented [33]. Similar approaches have been met with success in the treatment of HIV in PWID by integrating mental health or substance abuse treatment services with anti-retroviral treatment (ART) delivery [49].

We found that positive support from clinic staff, especially peer counselors, facilitated HCV treatment adherence among PWID. Patients stated interferon-based regimens and side effects were more tolerable when they felt respected by doctors and nurses [29, 37]. Peer counselors acting as a bridge between patient and provider help to create a more comfortable setting to share information [32]. With regards to HIV treatment for PWID, peer counseling at the point of ART delivery has been associated with a 95 % treatment adherence rate among IDUs [50]. We anticipate that even in the advent of DAA therapy, peer counseling and positive support from clinic staff will continue to be an important facilitator of treatment adherence.

Our findings suggested that integration of HCV treatment services and mental health services facilitated HCV treatment adherence. Quantitative reviews addressing this topic evaluated studies using both pharmaceutical and behavioral interventions [45, 46] but have shown inconclusive results. Our qualitative data showed that patients view depression and mental distress as a significant barrier to continuing treatment. Individuals living with HCV have an increased prevalence of mental illness because of virus-mediated effects, [51] treatment-related side effects, and comorbidities common among PWID [16]. Untreated mental health problems in turn can lead to a vicious cycle of poor adherence because of low mood, poor concentration and anxiety [16]. Integration of HCV treatment and mental health services may improve both SVR and mental health outcomes, [46] though more research is required. Given hesitation on the part of some providers to treat individuals with HCV and ongoing mental health problems, integration of services may also help expand access to HCV treatment among PWID [52].

### Moving towards the age of universal DAA treatment regimens

As DAAs are a relatively new development, the literature reviewed in our study all dealt with treatment regimens involving interferons and all of their associated side effects. While there is a move towards DAAs as the standard of care (as is the case in the United States, the United Kingdom, Australia and other high-income countries), we predict there will still be some time until they are universally adopted. While price is the most significant barrier for many countries, other issues such as drug registration and approval may also delay the process [53].

### Limitations

Our study has several limitations. Nine out of the ten studies examined individuals receiving interferon-based treatment. The only study including patients on DAA therapy still included pegIFN + RBV in the triple therapy regimen [34]. Interferon-free DAA regimens have fewer side effects, [54] and so our findings directly related to inability to cope with side effects, withdrawal-like side effects and injections reminiscent of drug use will be less of an issue with interferon-free DAA therapy. However, several of the themes identified are still relevant, including the importance of HCV treatment service integration, nonjudgmental clinic staff, flexible clinic hours, and an understanding of the PWID identity. Another major limitation of our study was that all include studies were conducted in high-income, English-speaking countries, despite the search being conducted with no language restrictions. It is concerning that there is a lack of available literature on the topic from low- and middle-income countries. However, this is consistent with the fact that there are decreased rates of HCV testing and treatment in these setting as well [55]. While it is difficult to generalize our findings to lower-income settings with any certainty, similar reviews conducted on facilitators and barriers of HIV treatment adherence in developing and developed countries have found several areas of overlap. While themes directly relating to access to treatment and financial difficulties are more prevalent in developing countries, more patient-centered themes such as fear of stigma, mistrust of the medical establishment and concurrent substance addiction and mental illness remained equally relevant in both developing and developed countries [56]. We expect that the same would be true of our findings.

## Conclusion

Our review highlights the need for more qualitative research on hepatitis C service delivery among PWID. As DAA use expands, further qualitative evaluation will be essential to ensure that HCV service delivery models are responsive to the unique needs and preferences of PWID. We anticipate that while some themes we identified will be less relevant with the gradual phasing out of interferon-based regimens, many of the core themes that we uncovered will not change. In particular, the need for integration of addiction and mental health services within HCV treatment delivery is unlikely to be affected by the introduction of DAAs. Some of the evidence gathered from delivering HIV services to PWID [57] may be able to inform the development of high quality HCV services for PWID.

## Additional files

Additional file 1: Electronic search algorithm for systematic review. (DOCX 107 kb) Additional file 2: Primary source quotations. (DOCX 97 kb)

## Abbreviations

ART: Anti-retroviral ‘’
DAA: Direct acting antivirals
HCV: Hepatitis C virus
HIV: Human immunodeficiency virus
IVDU: Intravenous drug use
pegIFN + RBV: pegylated interferon and ribavirin
PWID: People who inject drugs
SVR: Sustained virological response
