# Supplementary material for: Facilitators of HCV treatment adherence among people who inject drugs: a systematic qualitative review and implications for scale up of direct acting antivirals
Source: BMC Public Health. 2016 Sep 20;16:994. doi: 10.1186/s12889-016-3671-z (PMC5029046; doi:10.1186/s12889-016-3671-z)
Supplement: Additional file 1: — Electronic search algorithm for systematic review. (DOCX 107 kb) [file 12889_2016_3671_MOESM1_ESM.docx]

**Supplement 1. Electronic search algorithm for systematic review**

| Database | |  |
| --- | --- | --- |
| CINAHL | | |
| 5 | S1 OR S2 OR S3 OR S4 | Search modes - Boolean/Phrase |
| S4 | TX hepatitis c AND TX attitudes AND TX methadone OR TX intravenous drug use AND TX medication compliance | Search modes - Boolean/Phrase |
| S3 | TX hepatitis c AND TX perceptions AND TX methadone OR TX intravenous drug use AND TX medication compliance | Search modes - Boolean/Phrase |
| S2 | TX hepatitis c AND TX perceptions AND TX methadone OR TX intravenous drug use AND TX treatment adherence | Search modes - Boolean/Phrase |
| S1 | TX hepatitis c AND TX attitudes AND TX methadone OR TX intravenous drug use AND TX treatment adherence | Search modes - Boolean/Phrase |
| PubMed | | |
| hepatitis c AND "health knowledge, attitudes, practice" AND (intravenous drug abuse OR methadone) AND (treatment OR therapy OR antiviral OR interferon)  ("hepatitis c"[MeSH Terms] OR "hepatitis c"[All Fields] OR "hepacivirus"[MeSH Terms] OR "hepacivirus"[All Fields]) AND "health knowledge, attitudes, practice"[All Fields] AND (("substance abuse, intravenous"[MeSH Terms] OR ("substance"[All Fields] AND "abuse"[All Fields] AND "intravenous"[All Fields]) OR "intravenous substance abuse"[All Fields] OR ("intravenous"[All Fields] AND "drug"[All Fields] AND "abuse"[All Fields]) OR "intravenous drug abuse"[All Fields]) OR ("methadone"[MeSH Terms] OR "methadone"[All Fields])) AND (("therapy"[Subheading] OR "therapy"[All Fields] OR "treatment"[All Fields] OR "therapeutics"[MeSH Terms] OR "therapeutics"[All Fields]) OR ("therapy"[Subheading] OR "therapy"[All Fields] OR "therapeutics"[MeSH Terms] OR "therapeutics"[All Fields]) OR antiviral[All Fields] OR ("interferons"[MeSH Terms] OR "interferons"[All Fields] OR "interferon"[All Fields])) | | |
| PSYCInfo | | |
| su(hepatitis c) AND (treatment OR interferon OR therapy OR antiviral) AND (methadone OR intravenous drug use or injection drug use) AND (attitudes OR perceptions OR adherence OR compliance) | | |
