# Supplementary material for: Facilitators of HCV treatment adherence among people who inject drugs: a systematic qualitative review and implications for scale up of direct acting antivirals
Source: BMC Public Health. 2016 Sep 20;16:994. doi: 10.1186/s12889-016-3671-z (PMC5029046; doi:10.1186/s12889-016-3671-z)
Supplement: Additional file 2: — Primary source quotations. (DOCX 97 kb) [file 12889_2016_3671_MOESM2_ESM.docx]

**Supplement 2. Primary source quotations**

| **Theme 1: Logistical Support for Patients** | | | |
| --- | --- | --- | --- |
|  | | **Support outside the clinic** | |
|  | | 1 | *“Well partly the transport [is a barrier], but I think it’s a psychological thing for a lot of our patients, they’re very entrenched in their own environment. You talk to a lot of patients here who’ve lived all their lives in the East End and they don't know the West End… that's a foreign land to them it really is. You tell them that you've got an appointment at [hospital name], well you might as well say it’s in Timbuktu because they have no idea where that is*.” [Nurse][[29](#_ENREF_29)] |
|  | | 2 | *“It does make [HCV treatment] hard because you’re sleeping on the streets and you’re getting up at all hours of the night, so you’re not getting much sleep. And you get a bit aggravated and you get told that ‘You can’t sleep here, you can’t sleep there, you can't go there, you cant go here*.’” [40 year old male] [[37](#_ENREF_37)] |
|  | | 3 | *“I wouldn't think of doing this if I was out on the street. Not taking something that makes you as violently sick as that stuff does. I mean I used to be in bed after taking that stuff, just shaking. Water pouring off of me, no matter how many covers you put on me, I still felt cold. I used to rattle the whole room. I would never been able to go through that out on the street.”* [[12](#_ENREF_12)] |
|  | | **Accommodating clinic** | |
|  | | 4 | *“My needs are met in a whole lot of different ways, from personal to support, to my addiction to ramifications from the addiction. And um, there is a GP here as well… you can be treated for other stuff.”*[[35](#_ENREF_35)] |
|  | | 5 | *“It’s no use… you can only go if you've got an appointment but how do you know that in two weeks time at 11:30 a.m. you are going to have a problem? Life’s not like that.”* [Female] [[29](#_ENREF_29)] |
| **Theme 2: Positive interactions with support system** | | | |
|  | | **Compassionate Providers** | |
|  | | 7 | *“I had the feeling that even for my nurse and doc this was not routine. Then I realized they were fighting along with me. I really absolutely knew: I suffer from something they don’t really know about. But let’s say, at the worst point, both of them stood with me.” [*[*39*](#_ENREF_39)*]* |
|  | | **Support of current and former injection drug users** | |
|  | 8 | | *“And for some people it might be easier to talk to someone like [the peer support worker] rather than the doctors so you still get the information across.”* [[35](#_ENREF_35)] |
| **Theme 3: Understanding drug user identity** | | | |
|  | **Avoiding relapse and managing ongoing drug use** | | |
|  | | 9 | *“It’s got a syringe thing on it, you can pull it out, and that triggers me. I get a recall just from holding it and everything, and when I’m flushing and washing it out, I’m [thinking] ‘you’re getting a bit too into this.’”*[41 year-old female][[28](#_ENREF_28)] |
|  | | 11 | *“But certainly there are a few of the strategies that patients use that help us with [managing side effects]. Quite a few patients smoke cannabis and cannabis smoking is very common (laughs). You know, I don’t recommend patients smoke cannabis but they often do for relief of nausea, to help them sleep, to help with aches and pains, to help them relax. So, if they continue to do that, that’s fine. I just ask them to tell me how much they’re doing it and, if they increase it a lot, because it can impact on mood . . . I guess those sorts of things are what patients come back and tell me about.”* [Healthcare provider] [[30](#_ENREF_30)] |
